# Using Oxidized Low-Density Lipoprotein Autoantibodies to Predict Restenosis after Balloon Angioplasty in Patients with Acute Myocardial Infarction

**DOI:** 10.1371/journal.pone.0074726

**Published:** 2013-10-03

**Authors:** Ching-Hui Huang, Chia-Chu Chang, Ching-Shan Huang, Chen-Ling Kuo, Ching-Pei Chen, Chien-Hsun Hsia, Yung-Ming Chang, Hung-Te Chen, Chih-Chung Feng, Lee-Shin Lin, Po-Ta Yang, Chen-Dao Tsai, Chih-Sheng Lin, Chin-San Liu

**Affiliations:** 1 Division of Cardiology, Department of Internal Medicine, Changhua Christian Hospital, Changhua, Taiwan; 2 Department of Biological Science and Technology, National Chiao Tung University, Hsinchu, Taiwan; 3 Division of Nephrology, Department of Internal Medicine, Changhua Christian Hospital, Changhua, Taiwan; 4 School of Medicine, Chung Shan Medical University, Taichung, Taiwan; 5 Vascular and Genomic Research Center, Changhua Christian Hospital, Changhua, Taiwan; 6 Department of Neurology, Changhua Christian Hospital, Changhua, Taiwan; 7 Graduate Institute of Integrative Medicine, China Medical University, Taichung, Taiwan; University of Missouri, United States of America

## Abstract

**Objectives:**

Oxidized low-density lipoproteins (oxLDL) and oxidized low-density lipoprotein autoantibodies (OLAB) have been detected in human plasma and atherosclerotic lesions. OLAB appear to play a role in the clearance of oxLDL from circulation. Higher levels of OLAB appear to be associated with a reduced risk of a wide range of cardiovascular diseases. We investigated the prognostic value of plasma oxLDL and OLAB in patients undergoing primary coronary balloon angioplasty for acute ST-elevation myocardial infarction (STEMI).

**Methods:**

Plasma oxLDL and OLAB concentrations were measured in 56 patients with acute STEMI before primary angioplasty, and then 3 days, 7 days and 1 month after the acute event. Follow-up angiography was repeated 6 months later to detect the presence of restensosis (defined as >50% luminal diameter stenosis). The thrombolysis in myocardial infarction (TIMI) risk score was calculated to determine the relationship between OLAB/oxLDL ratio and TIMI risk scores.

**Results:**

Of the 56 patients, 18 (31%) had angiographic evidence of restenosis. Plasma OLAB concentrations were significantly lower in the restenosis group before angioplasty (181±114 *vs*. 335±257 U/L, *p* = 0.003), and at day 3 (155±92 *vs.* 277±185 U/L, *p*<0.001) and day 7 (177±110 *vs.* 352±279 U/L, *p*<0.001) after the acute event. There was no difference in oxLDL concentration between the two groups. The ratio of OLAB/oxLDL positively correlated with TIMI risk scores before angioplasty (*p* for trend analysis, *p* = 0.004), at day 3 (*p* = 0.008) and day 7 (*p*<0.001) after STEMI.

**Significance:**

A relative deficit of OLAB, and hence likely impaired clearance of oxLDL, is associated with the risk of arterial restenosis after primary angioplasty for acute STEMI.

## Introduction

The main cause of acute myocardial infarction (AMI) is the rupture of vulnerable plaques and the subsequent formation of thrombi [1]. The lipid component of vulnerable plaques is highly enriched with oxidized low-density lipoproteins (oxLDL) [2], which are released from atherosclerotic plaques into the circulation on rupture [3]. Circulating oxLDL is atherogenic and immunogenic, thereby triggering innate and humoral immune responses [4]. As oxidized low-density lipoprotein autoantibodies (OLAB) play a role in the clearance of oxLDL from the circulation, it has been hypothesized that they may protect against the risk of atherosclerosis [5]. However, whether OLAB is proatherogenic or antiatherogenic has not been established *in vivo*.

The presence of circulating OLAB may, however, simply reflect the oxidative burden of low-density lipoprotein cholesterol (LDL-C). It is also possible that circulating OLAB might directly or indirectly contribute to atherogenesis [6]. In our previous study of healthy volunteers, we found that a low ratio of serum OLAB to oxLDL concentration was associated with thickening of the intima of the carotid artery and elevated serum LDL concentration. Conversely, subjects with a high OLAB to oxLDL ratio had the lowest carotid artery intimal thickness (CCA-IMT) and lowest LDL levels. These findings suggest that both a high plasma oxLDL and low plasma OLAB contribute to the development of atherosclerosis [7]. Pawlak et al recently found that the ratio of serum OLAB to oxLDL influenced the extent of carotid atherosclerosis and risk of cardiovascular complications in dialyzed patients with renal impairment [8], leading the authors to hypothesized that the ratio reflects the balance between the modification of LDL induced by oxidative stress and the rate of clearance of oxLDL from the circulation. However, the hypothesis remains to be explored.

In this study, therefore, we hypothesized that the ratio of circulating OLAB to oxLDL (OLAB/oxLDL ratio) concentration may be associated with an atheroprotective phenotype and that thrombolysis in myocardial infarction (TIMI) risk score, a well-known prognostic marker [9], [10], may be associated with the OLAB/oxLDL ratio. Certain promising biomarkers have been shown to provide prognostic information about restenosis in patients undergoing balloon angioplasty for AMI [11]. The use of biomarkers in the early detection of patients at high risk of restenosis may be clinically relevant as it may help identify patients who might benefit from targeted pharmaceutical intervention or alternative therapies to reduce restenosis.

## Methods

### Subjects and study protocol

This was a prospective study of 65 consecutive patients with a *de novo* ST elevation AMI who underwent primary percutaneous coronary intervention (PCI) and thromboaspiration between May 2009 and May 2010. Nine patients who underwent direct stenting were excluded, but the other 56, who underwent a balloon angioplasty, were enrolled into the study. During the study period, guidelines stated that the primary aim of PCI was to achieve revascularization without direct stenting [12]. Venous blood was obtained prior to PCI and at day 3, day 7 and 1 month after the acute event. Blood sampling was undertaken after an 8-hour fast, with the exception of the sample taken prior to PCI. Diagnosis of STEMI was primarily based on the Joint Taskforce universal definition of myocardial infarction [13]. The diagnostic criteria used were: ST segment elevation of >0.2 mV in two or more contiguous electrocardiography (ECG) leads and an increase in cardiac biomarkers (for example troponin I and creatinine kinase (CK) MB fraction) with at least one value above the 99^th^ percentile of the upper reference limit within 24 hours of the onset of pain. The culprit vessel was identified based on clinical, ECG and angiographic findings. All patients were placed on aspirin and clopidogrel prior to PCI, which is the standard regimen in Taiwan. Angiography was repeated for patients who developed angina within 6 months, or after 6 months in asymptomatic patients. The TIMI risk score was calculated for all patients. It was calculated as the weighted sum of several clinical predictors including age ≥75 years (3 points); age 65 to 74 (2 points); history of angina, diabetes, or hypertension (1 point); Killip class II to IV (2 points); heart rate >100 beat/min at the time of presentation (2 points); systolic blood pressure <100 mm Hg at the time of presentation (3 points); anterior myocardial infarction or left bundle branch block (1 point); time to treatment >4 hours from symptom onset (1 point); and weight <67 kg (1 point) [9]. Data obtained from the AMI group included age, sex and the presence of risk factors (for example cigarette smoking, diabetes mellitus, hypertension and hypercholesterolemia), clinical variables and medication history. Smoking index was defined as the number of packs smoked per day × years smoked. The protocol was approved by the Institutional Review Board of the Changhua Christian Hospital, Taiwan, and all subjects gave written and informed consent to participate.

### Measurement of plasma biochemical parameters

The plasma oxLDL concentration was determined by a competitive enzyme-linked immune-absorbent assay (ELISA) [14] with a specific murine monoclonal antibody, mAb-4E6 (Mercodia, Sylveniusgatan, Sweden). The plasma OLAB concentration was measured using a specific ELISA kit (Biomedica, Wein, Austria). The assay was performed according to the manufacturers' instructions.

Plasma total cholesterol, high-density lipoprotein cholesterol (HDL-C), and triglyceride levels were determined using an enzymatic technique as previously described [15]. LDL-C concentration was calculated according to the formula developed by Friedewald *et al*. [16], which is as follows: LDL-C  =  (total cholesterol – high-density lipoprotein-C-triglyceride)/5.

### Angiographic assessment

Quantitative coronary angiographic measurements were made by a cardiologist blinded to the patients' OLAB/oxLDL status. Restenosis was defined as a >50% diameter stenosis on a repeat angiography at or within 6 months of the acute event. The TIMI flow [17] and myocardial blush [18] grades for assessing microvascular perfusion were also reviewed by the same cardiologist. Characteristic coronary lesions were classified according to the American College of Cardiologists/American Heart Association classification [19].

### Statistical analyses

Statistical analyses were undertaken using the SPSS statistical software package (SPSS v15.0; Chicago, IL, USA). All parameters are presented as the mean with the standard deviation. Statistical differences between the subgroups with and without restenosis within the AMI group were evaluated using the Mann-Whitney U test. A *p* value <0.05 was considered statistically significant. A Spearman's rho correlation was used to analyze the relationships between OLAB and patient characteristics. Receiver operator characteristic (ROC) curves were constructed to assess the predictive accuracy of OLAB for restenosis. The areas under the curves (AUC) for predicting restenosis with OLAB were calculated. A general linear model technique was used to evaluate independent associations between OLAB and other measured variables. The Jonckheere-Terpstra test was used to analyze the association between the OLAB/oxLDL ratio and TIMI risk scores. The Jonckheere-Terpstra test is similar to the Kruskal–Wallis test but is applied to samples with a priori ordering, e.g., TIMI risk scores. When there is an a priori ordering, the Jonckheere test has more statistical power than the Kruskal–Wallis test. Since our study was a pilot study, there was no reference data for OLAB in AMI patients. Therefore, it was difficult to estimate sample size and study power.

## Results

### Baseline clinical characteristics, biochemical data, medication history, and angiographic characteristics of patients with AMI

The AMI group was divided into two subgroups based on the presence or absence of restenosis (a ‘No-Restenosis’ group and ‘Restenosis’ group) in the 6 months after the acute event. Restenosis was evident on angiography in 18 patients (31%). Their baseline characteristics are summarized in **Table 1**. Serum OLAB concentration was significantly lower in the Restenosis group than in the Non-Restenosis group. There were no significant differences in the cardiac muscle enzyme level, lipid profile, location of the infarct-related artery, ECG findings, lesion characteristics, or quality of initial reperfusion.

**Table 1:** Baseline clinical, biochemical and angiographic characteristics of the study population.

	Non-restenosis	Restenosis	*P* value
	(n = 38)	(n = 18)	
Age (years)	57.5±12.5	57.4±10.4	0.984
Gender (male/total)	23/38	12/18	0.054
Risk factors			
Hypertension (%)	71	83	0.301
Dyslipidemia (%)	68	83	0.251
Diabetes mellitus (%)	24	33	0.477
Smoking (%)	61	50	0.804
Cholesterol (mg/dL)	192±45	196±53	0.605
HDL-C (mg/dL)	41±10	43±10	0.434
LDL-C (mg/dL)	136±37	138±46	0.551
Triglycerides (mg/dL)	94±140	90±78	0.792
hsCRP (mg/dL)	0.388±1.044	0.517±0.784	0.317
Fasting glucose (mg/dL)	179±114	170±49	0.226
Metabolic syndrome (%)	97	61	0.385
CPK (maximum) (U/L)	2,461±2,262	2,221±1,574	0.834
CKMB (maximum) (ng/mL)	224±185	271±210	0.557
Troponin I (ng/mL)	5.63±17.9	1.71±4.13	0.274
oxLDL (mg/dL)	68.3±21.9	66.0±20.0	0.599
OLAB (U/L)	335±257	181±114	0.012
OLAB/oxLDL	6.11±6.69	3.17±2.07	0.016
Echocardiographic Parameters			
Ejection fraction (%)	60±12	63±9	0.230
WMSI	1.27±0.21	1.29±0.17	0.576
Infarct-related coronary artery			0.234
LAD (%)	57	44	
LCX (%)	11	17	
RCA (%)	32	39	
Medications			
Antiplatelet (%)	100	100	−
ACEI/ARB (%)	90	89	0.949
Beta-blocker (%)	29	44	0.283
Statin (%)	82	83	0.875
Myocardial blush grade	2.08±0.87	1.89±0.93	0.468
Lesion length (mm)	18.7±7.6	20.8±5.5	0.296
Pre-PCI TIMI flow grade	0.66±1.07	0.44±0.92	0.471
Post-PCI TIMI flow grade	2.97±0.16	2.94±0.23	0.590
Lesion calcification	0.24±0.59	0.17±0.38	0.648
Lesion complexity	1.00±0.52	0.83±0.51	0.266
TIMI risk score	2.98±1.15	2.50±1.04	0.171

Follow-up angiography was repeated 6 months later to detect the presence of restenosis (defined as >50% luminal diameter stenosis).

HDL-C  =  high-density lipoprotein cholesterol; LDL-C  =  low-density lipoprotein cholesterol; hsCRP  =  high-sensitivity C-reactive protein; CPK  =  creatine phosphokinase; CKMB  =  creatinine kinase MB fraction; oxLDL  =  oxidized low-density lipoprotein; OLAB  =  oxidized low-density lipoprotein autoantibodies; WMSI  =  wall motion score index; LAD  =  left anterior descending artery; LCX  =  left circumflex artery; RCA  =  right coronary artery; ACEI  =  angiotensin-converting enzyme inhibitors; ARB  =  angiotensin II receptor blockers; TIMI  =  thrombolysis in myocardial infarction; PCI  =  percutaneous coronary intervention.

### Serial circulating oxLDL levels in patients with and without restenosis

**Figure 1:**
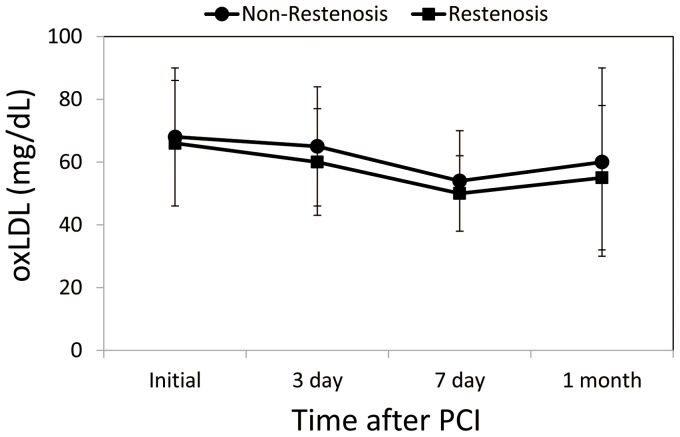
Changes in oxidized low-density lipoprotein (oxLDL) levels in patients in the Restenosis and Non-Restenosis groups. The patients received follow-up angiography 6 months after PCI to detect the presence of restensosis (n = 18) or non-restenosis (n = 38). Restenosis is defined as >50% luminal diameter stenosis.

There were no significant differences in oxLDL levels before PCI (66±20 mg/dL *vs*. 68±22 mg/dL, *p* = 0.716), on day 3 (60±7 mg/dL *vs*. 65±19 mg/dL, *p* = 0.326), day 7 (50±12 mg/dL *vs*. 54±16 mg/dL, *p* = 0.348), or 1 month (55±23 mg/dL *vs*. 60±30 mg/dL, *p* = 0.541) after the acute event (**Figure 1**).

### Serial circulating OLAB levels in patients with and without restenosis

**Figure 2:**
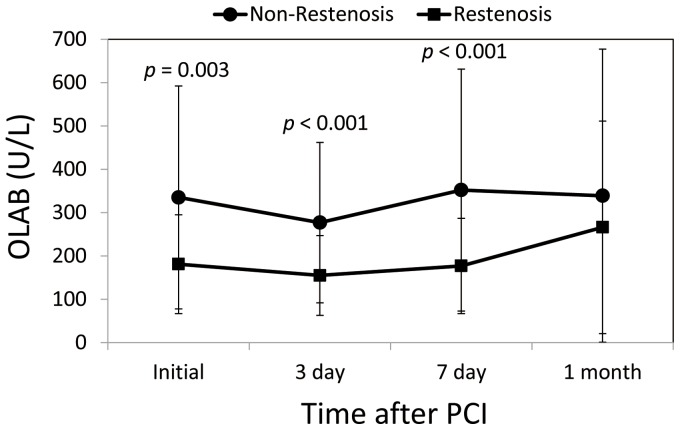
Changes in oxidized low-density lipoprotein autoantibodies (OLAB) levels in patients in the Restenosis (n = 18) and Non-Restenosis (n = 38) groups. ** *p*<0.01 and *** *p*<0.001, Mann-Whitney U test.

Compared with patients without restenosis, OLAB levels were significantly lower in those who developed restenosis, before PCI (181±114 U/L *vs*. 335±257 U/L, *p* = 0.003), on day 3 (155±92 U/L *vs*. 277±185 U/L, *p*<0.001), day 7 (177±110 U/L *vs*. 352±279 U/L, *p*<0.001), and at 1 month (266±245 U/L *vs*. 339±340 U/L, *p* = 0.416) after the acute event (**Figure 2**).

### Serial ratios of OLAB/oxLDL in patients with and without restenosis

Compared with patients without restenosis, the ratio of OLAB to oxLDL was significantly lower in those who developed restenosis before PCI; however, there was no significant difference in ratio between the groups 3 days, 7 days or 1 month after the acute event (**Table 2**).

**Table 2:** Serial ratios of OLAB/oxLDL in the patients with and without restenosis.

	Non-restenosis	Restenosis	*P* value
	(n = 38)	(n = 18)	
OLAB/oxLDL baseline	7.24±9.56	3.16±2.07	0.016
OLAB/oxLDL day 3	5.73±6.48	3.03±2.21	0.093
OLAB/oxLDL day 7	7.28±6.42	4.06±3.04	0.053
OLAB/oxLDL 1 month	6.11±6.03	7.06±9.19	0.646

oxLDL  =  oxidized low-density lipoprotein; OLAB  =  oxidized low-density lipoprotein autoantibodies.

### Prognostic value of serial OLAB levels in AMI patients after primary balloon angioplasty

**Figure 3:**
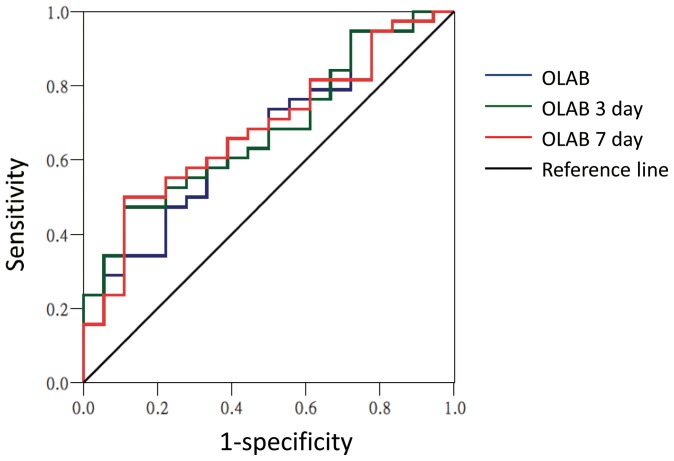
Prognostic value of serial oxidized low-density lipoprotein autoantibodies (OLAB) for restenosis in STEMI patients after primary balloon angioplasty. The areas under the curves (AUC) for OLAB as a predictor of restenosis were 0.697 before PCI (95% CI, 0.557–0.838; *p* = 0.018), 0.708 on day 3 (95% CI, 0.570–0.845; *p* = 0.013), and 0.712 on day 7 (95% CI, 0.576–0.848; *p* = 0.011).

The areas under the curves (AUC) for OLAB as a predictor of restenosis were 0.697 before PCI (95% CI, 0.557–0.838, *p* = 0.018), 0.708 on day 3 (95% CI, 0.570–0.845, *p* = 0.013), and 0.712 on day 7 (95% CI, 0.576–0.848, *p* = 0.011) (**Figure 3**).

### The relationship between TIMI risk score and the OLAB/oxLDL ratio

Trends analysis showed that the ratio of OLAB to oxLDL was positively correlated with the TIMI risk score in the subacute stages, namely at baseline, at 3 days and 7 days after the acute event (*p* = 0.004, *p* = 0.008 and *p*<0.001, respectively); however, this relationship was no longer evident 1 month after the acute event of STEMI (**Table 3**).

**Table 3:** Trend analysis for the ratio of OLAB/oxLDL and TIMI risk score at different time points after acute myocardial infarction.

TIMI risk score	1	2	3	≥4	*P* value
**Initial**	2.30 (1.20–2.51)	2.27 (1.61–5.80)	4.78 (2.49–10.10)	4.81 (2.48–7.31)	0.004
**3 day**	1.88 (1.44–2.19)	2.30 (1.62–6.37)	3.74 (1.45–7.51)	3.96 (2.77–9.16)	0.008
**7 day**	2.10 (1.86–3.45)	3.14 (2.16–6.91)	6.01 (2.43–9.66)	6.67 (4.01–10.29)	<0.001
**1 month**	3.31 (1.90–6.63)	4.10 (2.71–8.02)	3.07 (2.45–8.67)	3.33 (2.90–14.29)	0.472

The OLAB/oxLDL ratio associated with each TIMI risk score was presented as a median value (interquartile range; i.e., 25^th^–75^th^ percentile). The Jonckheere-Terpstra test for trend was performed and *p* values were indicated.

The TIMI risk score was calculated as the weighted sum of several clinical predictors, including age ≥75 years (3 points); age 65 to 74 (2 points); history of angina, diabetes, or hypertension (1 point); Killip class II to IV (2 points); heart rate >100 beat/min at the time of presentation (2 points); systolic blood pressure <100 mmHg at the time of presentation (3 points); anterior myocardial infarction or left bundle branch block (1 point); time to treatment >4 hours from symptom onset (1 point); and weight <67 kg (1 point).

### Factors correlating with OLAB

The serum OLAB concentration negatively correlated with age, body mass index, waist circumference, oxLDL, LDL-C and total cholesterol, and positively correlated with high-density lipoprotein cholesterol (HDL-C) and TIMI risk score (**Table 4**).

**Table 4:** Univariate correlation between autoantibodies against oxidized low-density lipoprotein (OLAB) level and patient characteristics.

Variable	Rho correlation coefficient	*P* value
Age (years)	−0.363	<0.001
Body mass index (kg/m^2^)	−0.210	0.006
Waist circumference (cm)	−0.210	0.009
oxLDL (mg/dL)	−0.288	0.008
LDL-C (mg/dL)	−0.297	0.001
HDL-C (mg/dL)	0.209	0.006
Total cholesterol (mg/dL)	−0.260	0.006
TIMI risk score	0.424	<0.001

oxLDL  =  oxidized low-density lipoproteins; LDL-C  =  low-density lipoprotein cholesterol; HDL-C  =  high-density lipoprotein cholesterol; TIMI  =  thrombolysis in myocardial infarction.

### General linear model analysis of factors affecting OLAB

General linear model analysis was performed to examine the factors that are independently associated with OLAB in the AMI group. We found that the TIMI risk score <3 compared to TIMI risk score ≥3 showed a negative correlation with OLAB (*p* = 0.045) (**Table 5**).

**Table 5:** Results of general linear model by OLAB as dependent variable.

Parameter	B	SE	95% Confidence Interval		*P* value
			Lower	Upper	
Intercept	124.31	320.19	−525.06	773.66	0.700
Female *vs*. Male	−259.52	130.32	−523.81	4.77	0.054
CPK MB mass	0.081	0.297	−0.521	0.684	0.786
IRA-LAD *vs*. IRA-RCA	168.49	121.49	−77.91	414.89	0.174
IRA-LCX *vs*. IRA-RCA	144.45	216.18	−293.98	582.87	0.508
Age	4.509	4.749	−5.122	14.141	0.349
TIMI risk score <3 *vs*. ≥3	−242.55	116.60	−479.02	−6.08	0.045
D2B time	−0.784	1.342	−3.505	1.937	0.562

IRA  =  Infarct-related coronary artery; D2B  =  Door to balloon.

TIMI risk score <3 indicates low risk and ≥3 indicates intermediate to high risk.

## Discussion

In spite of the establishment of guidelines for secondary prevention of coronary artery diseases, many patients still develop restenosis after PCI. Therefore, circulating biomarkers that can help predict restenosis-prone conditions are necessary to improve the follow-up and treatment of the patients who have received PCI for coronary artery disease. In this study, we found that higher OLAB to oxLDL ratio was associated with an atheroprotective phenotype in patients who have undergone primary PCI for STEMI. To the best of our knowledge, this is the first study to comprehensively analyze the significance of circulating OLAB and oxLDL in STEMI. We found that OLAB/oxLDL ratio can be used to predict the likelihood of restenosis in patients who received PCI for STEMI.

In a previous study [7], we found that healthy volunteers with a low OLAB to oxLDL ratio had greater common carotid artery intima-media thickness (CCA-IMT) and higher LDL levels. Conversely, the subjects with a high OLAB to low oxLDL ratio had the smallest CCA-IMT and lowest LDL levels. These findings suggest that a high plasma oxLDL and low plasma OLAB contribute to the development of atherosclerosis. The same hypothesis has also been evaluated in uremic patients. Pawlak and colleagues [8] found that patients with no history of cardiovascular disease (CVD) undergoing renal dialysis had lower serum oxLDL and higher OLAB concentrations than dialyzed patients with CVD and that CCA-IMT was positively associated with oxLDL/OLAB ratio. Children and young adults, who typically are at very low risk of atherosclerosis, have significantly higher levels of circulating OLAB and very low levels of oxLDL compared with healthy older adults [20], [21]. Shoji *et al*. have also reported an inverse relationship between circulating oxLDL and OLAB levels in healthy subjects, which is consistent with our findings in our previous study [22].

OLAB binds oxLDL and, in turn, decreases circulating oxLDL levels [5]. Additionally, OLAB levels appear to be negatively correlated with indices of oxidative stress [23] and negatively associated with the severity and extent of stenotic coronary lesions [24]. Therefore, OLAB may protect against atherosclerosis by attenuating oxidative stress. We hypothesize that under normal physiological conditions, there is a dynamic balance between circulating oxLDL and OLAB, where OLAB prevents an increase in circulating oxLDL.

This hypothesis is based on the results of several studies. For example, apolipoprotein E (apoE)-deficient mice demonstrate an ectopic overexpression of liver lectin-like oxLDL receptor-1 (LOX-1), which in turn enhances the hepatic uptake of oxLDL [25]. As a result, these animals have decreased levels of plasma oxLDL and show a slower progression of atherosclerosis [25]. These findings clearly demonstrate that a lower level of oxLDL is beneficial in preventing atherosclerosis. Furthermore, it has been reported that treatment with recombinant antibodies against aldehyde-modified apoB-100, a component of oxLDL, reduces plasma levels of oxLDL by 34%, dramatically reduces atherosclerosis, and inhibits restrictive vascular remodeling in mice expressing human apoB-100 [26]. These observations support the notion that OLAB levels not only reflect the extent of atherosclerosis, but also play a role in maintaining lower levels of circulating oxLDL. Our study concurs with the conclusions of Pawlak and colleagues, who proposed that the OLAB to oxLDL ratio should be seen as a new biomarker that reflects the balance between *in vivo* oxidative stress burden and the clearance rate of oxLDL from the circulation [8]. Based on these findings, it is clear that measuring the levels of either oxLDL or autoantibodies alone is insufficient. Instead, clinicians should assess the reciprocal changes between circulating oxLDL and OLAB levels.

Within this cohort, oxLDL levels was similar between patients who developed restenosis and those who did not, allowing the direct impact of circulating OLAB levels on restenosis following primary balloon angioplasty to be examined without adjusting for oxLDL. We found that the prognostic value of OLAB in predicting restenosis within 7 days after STEMI is better than the OLAB/oxLDL ratio. As shown in **Table 2**, serial ratio was only significant before PCI. However, the OLAB levels at different time points differed significantly within 7 days after STEMI. We also found that the maximum area under the curves (AUC) for OLAB/oxLDL ratio was 0.682 at 7 days, which was less than the AUC for OLAB (0.712) (data not shown). A larger sample size is needed to determine the best cut-off value of OLAB in predicting restensosis in patients with STEMI who receive primary angioplasty. We found an inverse relationship between the concentration of circulating OLAB and several markers of atherosclerotic risk (i.e. body mass index, waist circumference, oxLDL, LDL, and age), which is in broad agreement with the findings reported by Che and colleagues [24]. Additionally, the serum concentration of OLAB was found to positively correlate with that of HDL-C.

There is also a temporal element to the importance of OLAB. For example, an early decrease in circulating anti-oxLDL antibodies has been shown to be associated with the severity of coronary artery disease after acute coronary syndrome in patients with metabolic syndrome [27]. We found a high concentration of oxLDL immediately after STEMI, which was presumably released into the circulation by plaque rupture and triggering inflammatory and immune responses, which were neutralized by OLAB after 3 days. Although OLAB levels gradually returned to baseline after the acute episode, circulating oxLDL subsequently remained at lower levels. Other treatments may also influence OLAB concentration, such as control of hypertension [26], and we found no significant difference in OLAB levels between the restenosis and non-restenosis groups after 1 month.

We also found that the ratio of OLAB to oxLDL positively correlated with TIMI risk score at baseline and on days 3 and 7, but not 1 month, after STEMI. The TIMI risk score is a simple and well-validated clinical risk score, which is used to predict 30 days to 5 years mortality after STEMI [9], [10]. It also may serve as a risk stratification tool to help clinicians choose the most appropriate therapy. Our findings suggest that a higher OLAB/oxLDL ratio might be protective against the adverse sequelae of the acute insult, highlighting the importance of the dynamic balance between oxidative stress burden and oxLDL clearance rate. General linear model analysis indicated that the association between OLAB and TIMI risk score was independent of other factors, including age, sex, and the extent of heart muscle injury and the location of the infarct related coronary artery. Therefore, STEMI patients with a TIMI risk score ≥3 but with a low OLAB/oxLDL ratio should receive aggressive therapeutic intervention to improve clinical outcome. Manipulating the ratio, either by elevating OLAB levels or decreasing oxLDL concentration, may provide a preventive treatment strategy.

The limitations of this study include the relatively small size of the cohort, and the fact that OLAB and oxLDL were only tested within 7 days and 1 month after the acute event. Therefore, studies with larger numbers of participants and blood samples taken 14 days and 21 days after STEMI are needed to further test our hypothesis. We also excluded patients with STEMI who underwent direct stenting; however, this provided us with the chance to study the influence of oxLDL and OLAB levels on restenosis in patients who have not received direct stenting. Further studies are warranted to clarify the molecular mechanisms that underpin the possible protective effects of autoantibodies against restenosis, including in patients receiving coronary stents.

## Conclusion

We found that patients with lower OLAB levels within 7 days after STEMI were significantly more likely to have restenosis in the infarct-related artery after primary angioplasty. The ratio of OLAB to oxLDL positively correlated with the TIMI risk score in the subacute stages of STEMI. Our data support the hypothesis that higher circulating OLAB/oxLDL ratio is associated with an atheroprotective phenotype and that it is a potential prognostic biomarker for post-STEMI complications.
